# Special Issue “Development, Characterization and Applications of Novel Polymeric Materials and Composites”

**DOI:** 10.3390/ijms27020936

**Published:** 2026-01-17

**Authors:** Xiao Hu

**Affiliations:** 1 Department of Physics and Astronomy, Rowan University, Glassboro, NJ 08028, USA; hu@rowan.edu; 2 Department of Biological & Biomedical Sciences, Rowan University, Glassboro, NJ 08028, USA

It is our pleasure to introduce this Special Issue (SI) on “Development, Characterization and Applications of Novel Polymeric Materials and Composites”, which brings together recent advances across polymer science, composite engineering, biotechnology, and emerging applications. This collection was assembled with the goal of strengthening interdisciplinary links among material synthesis, theoretical modeling, advanced characterization, and translational technologies [1,2]. As scientific fields continue to specialize, interdisciplinary perspectives are increasingly essential for transforming polymer research into meaningful societal impacts [1,2,3]. The contributions gathered here reflect this vision by addressing both fundamental discoveries and application-driven developments.

This Special Issue contains eight articles, including seven research articles (Contributions 1–7) and one perspectives article (Contribution 8). Together, they span sustainable composite design, antimicrobial wound-healing membranes, thermosensitive colloidal systems, electrochemical transport in polymer matrices, polymer–amyloid interactions, polymer-assisted chemotherapy, cerium-enhanced scaffolds, and next-generation concepts in 3D bioprinting. These contributions are introduced below.

The first contribution (Contribution 1) investigates polycaprolactone (PCL) composites modified with kaolin residue derived from mining waste, showing that small additions of residue significantly enhance crystallization behavior, stiffness, and thermal resistance. Through combined kinetic modeling and morphological characterization, the authors demonstrate the role of the residue as an effective nucleating agent, offering a sustainable approach to improving polymer performance.

The second contribution (Contribution 2) focuses on the development of doxycycline-loaded membranes composed of carboxymethylcellulose (CMC) and chitosan for the potential treatment of diabetic foot ulcers. The authors systematically evaluated the membranes’ mechanical properties, hemocompatibility, and antibacterial activity, demonstrating strong inhibition of pathogenic bacteria along with favorable physical characteristics. These results highlight the promise of biopolymer-based wound dressings as localized therapeutic systems for chronic wound care.

Contribution 3 presents a detailed study of thermosensitive nanospheres synthesized from N-isopropyl acrylamide (NIPA) and hydrophilic comonomers using surfactant-free precipitation polymerization. By tuning initiator chemistry, comonomer composition, and crosslinking density, the researchers reveal how molecular architecture governs particle diameter, electrokinetic potential, and temperature-responsive solubility. Their insights broaden our understanding of colloidal stability and tunability in smart polymeric systems.

In Contribution 4, the author compares diffusion behavior in polymeric electrolytes to that in monomeric solvents, employing microband electrochemical techniques under highly viscous conditions. The findings show clear deviations from classical Fickian diffusion in polymer-rich systems, whereas monomeric solvents of similar viscosity display predictable linear diffusion. This work highlights the complexity of transport processes within polymer matrices and underscores the need for revised theoretical models.

Contribution 5 examines the interactions between polyallylamine and amyloid β fibrils, revealing that the polymer binds to amyloid structures and alters their fluorescence signatures. The study also demonstrates that polymer-coated amyloids behave differently in common detection assays, emphasizing both the opportunities and analytical challenges associated with creating functional amyloid–polymer materials.

In Contribution 6, the authors investigate dextran-graft-PNIPAM (poly-N-isopropylacrylamide) star-like polymers as nanocarriers for cisplatin and doxorubicin, showing that polymer–drug complexes enhance cytotoxicity in both drug-sensitive and drug-resistant carcinoma cell lines. Physicochemical characterization and mechanistic cellular studies reveal improved drug uptake and modified intracellular activity, illustrating the potential of stimuli-responsive polymeric nanocarriers in cancer therapy.

Contribution 7 focuses on electrospun polycaprolactone (PCL) scaffolds incorporating cerium-containing inorganic phases, including cerium oxide, cerium-doped calcium phosphates, and cerium-containing bioglass. The resulting composite scaffolds exhibit tailored fiber morphology, strong bioactivity, antibacterial effects, and excellent biocompatibility with human osteoblasts. These attributes underscore their potential for bone tissue engineering and related biomedical applications.

This Special Issue concludes with a perspective article (Contribution 8), which introduces a conceptual framework for 3D bioprinting that preserves liver cell asymmetry, an essential feature for maintaining metabolic compartmentalization and tissue organization. The authors propose a printing strategy based on oriented multicellular complexes supported by basement membrane-like structures, offering forward-looking insights into the future of organ fabrication and advanced bioprinting technologies.

Together, the contributions in this Special Issue (SI) demonstrate the breadth and vitality of contemporary macromolecular research. They illustrate how innovations in synthesis, molecular design, characterization, and application development can collectively advance the science and engineering of polymeric materials. We extend our sincere thanks to all authors, reviewers, and editors for their dedication and contributions. We hope that this collection will inspire continued interdisciplinary research and foster new developments in the creation and application of advanced polymeric systems.
